# 

**Published:** 1908-03-21

**Authors:** 


CONTENTS.

| | PAGE |
|---|---|
| **Authority and Teaching** | 643 |
| **Changes in the Vaccination Laws** | 644 |
| **Annotations—** | |
| The Sleeping Sickness Commission—Socialism, Cancer, and Advertisement—Cheap Food for School Children | 645 |
| **Medical Opinion and Movement** | 646 |
| **Hospital Clinics—** | |
| Nasal Obstruction and Operation for Rectifying Obstructive Deformities of the Nasal Septum. By Andrew Wylie, M.D., C.M., Glasgow | 649 |
| **Medicine—** | |
| Acute Nephritis with Extreme Hæmaturia... | 653 |
| **Points in Surgery—** | |
| Some Diseases of the Male Genital System... | 655 |
| **Medico-Legal Points—** | |
| Musical Bruits and Accidents to Workmen... | 656 |
| **Mental Diseases—** | |
| Two Cases of Restlessness—I. | 657 |
| **Resident Medical Officers' Department—** | |
| Practical Hints for X-ray Work | 658 |
| **Tropical Diseases—** | |
| Trypanosomiasis | 658 |
| **"The Hospital" Medical Book Supplement.—No. V.** | 659 |
| **Hospital Administration—** | |
| Current Hospital Topics... | 663 |
| Miss Nightingale and the City of London | 664 |
| Social and Poor law Problems | 664 |
| News and Coming Events | 665 |
| **Editor's Letter-Box—** | |
| Clean or Pasteurised Milk | 666 |
| **Nursing Administration—** | |
| Fever Nursing | 667 |
| **The Graduates' Medical Schools** | 668 |

